# Self-reported cataracts in older adults in Ghana: sociodemographic and health related factors

**DOI:** 10.1186/1471-2458-14-949

**Published:** 2014-09-12

**Authors:** Alfred E Yawson, Edith M Ackuaku-Dogbe, Nana A Hagan Seneadza, George Mensah, Nadia Minicuci, Nirmala Naidoo, Somnath Chatterji, Paul Kowal, Richard B Biritwum

**Affiliations:** Department of Community Health, University of Ghana Medical School, College of Health Sciences, Room 46, Korle-Bu, P.O. Box 4236, Accra, Ghana; Public Health Unit, Korle-Bu Teaching Hospital, Korle-Bu, Accra, Ghana; Department of Surgery, Eye Unit, University of Ghana Medical School, College of Health Sciences, Korle-Bu, Accra, Ghana; National Council Research, Institute of Neuroscience, Padova, Italy; World Health Organization, Multi-Country Studies unit, Geneva, Switzerland; University of Newcastle Research Centre on Gender, Health and Aging, Newcastle, Australia

**Keywords:** Older adult, Cataracts, Eye care service, Subjective wellbeing, Ghana, Low income countries

## Abstract

**Background:**

Changes in function of sensory organs with increasing age have significant impact on health and wellbeing of older persons. This paper describes cataract, a chronic eye condition, self-reported among older adults in Ghana and the need for improving access to eye care services.

**Methods:**

This work was based on the World Health Organization’s multi-country Study on global AGEing and adult health (SAGE), conducted in six countries including Ghana. SAGE Wave 1 in Ghana was conducted in 2007–2008 in a nationally representative sample of 4278 older adults, ≥ 50 years. Data were obtained on sociodemographic and health factors related to self-reported cataracts in older persons in Ghana. Data were analysed using descriptive measures (frequencies and proportions), chi-square test for associations in categorical outcome measures, and logistic regression for predictors of cataracts with SPSS version 21.

**Results:**

Overall prevalence of self-reported cataracts among 4278 older adults in Ghana was 5.4%. Prevalence was proportionately higher for women (5.9%) than men (4.7%). Reported cataracts increased with age, among urban residents, in older adults living without partners and among those with the worse life satisfaction index. Older adults in lower income groups, poorly educated or living alone had difficulty seeking vision care services. Prevalence was 8.4% among persons with diabetes, 10.4% among hypertensives and 11.4% in persons with previous history of stroke. Among older persons who had ever used alcohol or tobacco, prevalence rates of reported cataracts were 5.7% and 4.9%, respectively. Logistic regression analysis indicated that increasing age, lower income status and self-reported hypertension were significantly associated with cataract among older adults in Ghana.

**Conclusions:**

Cataract is prevalent in older people in Ghana with approximately 1 in 20 people aged 50 years or older reporting a previous diagnosis of cataract. As cataract surgery is restorative, a public health approach on behavioural modification, well structured national outreach eye care services (for rural residents), inclusion of basic eye health services at sub-district levels, increased family support and national health insurance for older persons is indicated.

## Background

Cataract is the leading cause of blindness accounting for nearly 48% of blindness globally [1, 2]. It poses a substantial economic and public health burden especially in developing countries. According to a World Health Organization (WHO) report on global blindness, approximately 90% of global cataract is seen in developing countries and estimates that more than 82% of all blindness occurs in individuals aged 50 and older [2].

In a population based study in Ghana, the prevalence rates of cataract causing low vision or blindness in people aged 40 years and above was 9% in the Volta region [3] and 2% in people aged 30 years and above in the Wenchi area of the Brong Ahafo region [4], two of the ten regions in Ghana. The burden of cataracts is not limited to financial costs to society alone, older persons living with un-operated cataracts are likely to have significantly reduced quality of life due to low vision. Other co-morbid conditions in older persons further reduces their quality of life. Decreases in functional abilities may be attributed to other age-related processes but may actually be associated with the onset of cataract.

Some health, lifestyle and environmental conditions have been associated with the development of cataracts. Research has strongly associated diabetes, smoking and ultra violet light exposure with the development of age related cataract [5–9]. Other factors inconclusively implicated in the development of cataracts include body mass index [10, 11] and post menopausal decline in estrogen [12].

Aging is often accompanied by the cumulative impact of chronic disease, increased prevalence of disability, worsening health status and reduced quality of life among the growing number of older persons. Aging, in itself, has been associated with a decline in visual functioning, with associated decline in physical and mental functioning. A number of studies have demonstrated impact of cataract related visual impairment on quality of life [13, 14]. These studies, showed that worsening of general functioning, psychosocial and overall eyesight were associated with increased visual loss. Those with increased visual loss were more likely to report dissatisfaction with quality of life and had poorer self-rated health than persons with normal vision.

In addition, smoking, body-mass index, and exercise patterns in midlife and late adulthood have been suggested as predictors of disability in later years. For the average person, efforts to reduce modifiable health risks may result in a postponement of initial disability and decreased lifetime disability [15].

Current eye health services in Ghana are organized at three levels, national, regional and district [16]. The national and regional levels provide specialized eye care services through the teaching and regional hospitals. Basic eye health services at the district is provided through district hospitals by ophthalmic nurses (i.e. state registered general nurses who have additional training in ophthalmology). Unlike the general health delivery system with structures to provide health care at sub-district and community level [16], eye health services is limited to the district level. Sub-districts and communities (where most of the rural population reside) receive eye care services through outreach programmes and special mobile eye clinics.

The goal of this analysis is to describe the sociodemographic, health related factors and life satisfaction index in older adults with self-reported cataract in Ghana. It is aimed at providing guidance for national plans on eye care for older persons and to contribute to national policy on interventions to mitigate health related factors in the older adult population.

## Methods

SAGE Wave 1 was undertaken in Ghana in a partnership between the University of Ghana’s Department of Community Health, the Ministry of Health and WHO, as part of a multi-country longitudinal study to complement existing aging data sources and to inform policy and programmes [17, 18]. A nationally representative sample of respondents ≥ 50 years were interviewed on socio-demographic background, health risk factors, chronic conditions, well-being and health insurance coverage. Face-to-face interviews and data entry were undertaken between May 2007 and June 2008. Ethical approval for SAGE Wave 1 in Ghana was given by WHO’s Ethical Review Board and the University of Ghana Medical School Ethics and Protocol Review Committee. All study participants provided informed consent [17, 18].

### Measures

Demographic and socioeconomic variables included sex, age, marital status, highest educational level completed, health insurance status and household income levels. Access to health care in Ghana is through the National Health Insurance Scheme (NHIS), introduced in 2003 and operationalized in most public and private health facilities in 2005. Basic eye care services including cataract surgery is covered by the NHIS. Assessment of health insurance status of older persons was based on this[19]. Educational level was categorized as low (primary or basic school completed or less) and high (at least secondary school completed or higher).

### Chronic diseases

SAGE collected data on prevalent chronic diseases in older persons in Ghana. Data on four of these chronic conditions (cataract, diabetes mellitus, hypertension and stroke) were used in this analysis. For cataract, the question asked was: “ In the last 5 years, were you diagnosed with a cataract in one or both of your eyes (a cloudiness in the lens of the eye) by a health care professional?” Diagnosis of cataract in Ghana is done by a doctor or nurse trained in ophthalmology. SAGE Wave 1 in Ghana included operated and unoperated cataract within the self-reported diagnosis of cataract in the last 5 years.

The prevalence rates for the other chronic diseases were obtained through responses to the question “*Has a health care professional ever told you, you have…?"*. Though SAGE Wave 1 measured blood pressure, the analysis considered only self-reported hypertension due to the focus on self-reported ill-health among older persons in Ghana [17, 18].

### Tobacco use

Lifetime tobacco use was assessed with the question ‘Have you ever smoked tobacco or used smokeless tobacco?’ SAGE considered tobacco products such as cigarettes, cigars, pipes, chewing tobacco, or snuff. Details on how tobacco use was assessed is provided in published elsewhere [17, 18, 20].

### Alcohol use

Lifetime alcohol use was assessed from responses to the question ‘Have you ever consumed a drink that contains alcohol (such as beer, wine, spirits, etc.)?’ SAGE Wave 1 quantified alcohol (commercially available and home-brewed beverages) in "standard drink" units as recommended by the World Health Organization [17, 18, 20].

### Body mass index (BMI)

Was obtained from measured weight and height of respondents.

### Subjective wellbeing (SWB)

Well-being or life satisfaction was assessed through a multi-dimensional scale, the WHO Disability Assessment Schedule 2.0, including a question about satisfaction with life overall [17, 18]. SWB as a single item measure was based on the overall life satisfaction question. As in a similar analysis by Yawson et al., 2013, the responses to this question were categorized into satisfied (very satisfied and satisfied), indifferent (neither satisfied nor dissatisfied) and not satisfied (dissatisfied and very dissatisfied) [21].

### Wealth or income quintiles

Were derived from the household ownership of durable goods, dwelling characteristics and access to services (improved water, sanitation and cooking fuel) for a total of 21 assets. Wealth levels were generated through a multi-step process, where asset ownership was converted to an asset ladder, Bayesian post-estimation method used to generate raw continuous income estimates, and then income transformed into quintiles [17, 18, 20].

### Data analysis

Demographic and socio-economic variables such as age, location (urban/ rural), educational level, marital status (due to its relevance in social support and health access), and income levels were described using proportions. Chi-square tests of significance were used to compare distribution of cataract and demographic, socioeconomic, health risk and life satisfaction indices.

Binary logistic regression analysis was done to determine factors associated with cataract in the older persons. Ever been diagnosed with cataract was the binary dependent variable in the regression model. Independent variables used included age, sex, location (urban/rural), educational level, marital status, income quintile, health insurance status, self-reported chronic conditions (diabetes, hypertension and stroke), alcohol use, tobacco use, and subjective wellbeing. Decisions were based on adjusted odds ratio [AOR] and p-values at 95% confidence level. Data were analysed using SPSS version 21.

## Results

Overall prevalence of cataracts in the 4278 older adults in Ghana was 5.4%. Prevalence was proportionately higher in women (5.9%) than men (4.7%); though this difference was not statistically significant (p-value = 0.067). The prevalence of cataract clearly showed an age gradient: higher values were reported in the older age groups (from 2.1% in 50–59 age group to 9.5% in ≥ 70 years). Age differences in prevalence of cataract was statistically significant (p-value = 0.001). Urban residents reported statistically significant higher rates (6.6%) than rural residents (4.4%). Older adults living without a partner (separated/divorced/ widowed) reported significantly higher prevalence of cataract (6.6%), compared to those living with a partner (married/cohabiting) (4.3%) as shown in Table 1. Older adults with secondary school education or higher, had a higher prevalence compared to those with primary school completed or less. Older adults in high income groups (Q4 and Q5) reported significantly higher levels of cataract (6.6%) compared to those within low income group (Q1 and Q2) (4.3%). Interestingly, older adults on national health insurance reported significantly higher prevalence of cataract (7.1%) than those without insurance (4.2%).

**Table 1:** **Prevalence of self-reported cataracts in older adults in Ghana by demographic and socioeconomic characteristics, SAGE Wave 1**

Characteristics	Self reported cataract		X ^2^ (p-value)
	n (%)	N	
**Sex**			
Male	106 (4.7)	2249	3.35 (0.067)
Female	120 (5.9)	2029	
**Age group**			
50–59	35 (2.1)	1681	
60–69	59 (4.9)	1196	83.88 (0.001)
70+	133 (9.5)	1401	
**Residence**			
Urban	115 (6.6)	1749	9.46 (0.002)
Rural	111 (4.4)	2529	
**Marital status**			
Never married	4 (8)	54	
Living with partner (married/cohabiting)	104 (4.3)	2426	11.92 (0.003)
Living without partner (Separated/divorced/Widowed)	119 (6.6)	1798	
**Highest educational level**			
Primary school completed or less	162 (5.2)	3114	0.229 (0.632)
Secondary school and above	66 (5.7)	1164	
**Income quintile**			
Q1 and Q2 (low)	105 (4.3)	2441	
Q3 (middle)	2 (5.7)	39	10.71 (0.005)
Q4 and Q5 (high)	119 (6.6)	1798	
**Health insurance**			
Yes	116 (7.1)	1638	16.58 (0.001)
No	111 (4.2)	2640	
**Total respondents**	231 (5.4)	4278	

As demonstrated in Table 2, prevalence of cataract among obese older adults was 5.8%. Among older adults with self-reported chronic non-communicable conditions, prevalence was 8.4% among those with diabetes, 10.4% among hypertensives and 11.4% in those with previous history of stroke. The prevalence of cataract among older adults who had ever used alcohol was 5.7%. Interestingly, older persons who had used tobacco had a lower prevalence of self-reported cataract (4.9%) compared to those who had never used tobacco.

**Table 2:** **Prevalence of self-reported cataracts in older adults in Ghana by health related factors and subjective wellbeing, SAGE Wave 1**

Characteristics	Self reported cataract		X ^2^ (p-value)
	n (%)	N	
**Obese**			
Yes	26 (5.8)	449	0.42 (0.519)
No	195 (5.1)	3829	
**Self-reported diabetes**			
Yes	14 (8.4)	162	3.31 (0.069)
No	214 (5.2)	4116	
**Self-reported hypertension**			
Yes	60 (10.4)	580	35.63 (0.001)
No	166 (4.5)	3698	
**Self-reported stroke**			
Yes	12 (11.4)	108	8.74 (0.003)
No	213 (5.1)	4170	
**Alcohol use**			
Ever	143 (5.7)	2512	0.001 (0.976)
Never	93 (5.3)	1766	
**Tobacco use**			
Ever	54 (4.9)	1111	0.395 (0.530)
Never	171 (5.4)	3167	
**Level of subjective well being/life satisfaction**			
Satisfied	113 (4.7)	2407	
Indifferent	105 (5.8)	1799	5.76 (0.056)
Not satisfied	7 (9.9)	72	

Analysis of satisfaction with daily living among older adults found the highest prevalence of cataract among those who expressed dissatisfaction with life (9.9%) and the lowest prevalence (4.7%) among those satisfied with life.

### Important associated factors of cataract in older adults in Ghana

Logistic regression analysis indicated that increasing age, income status and self-reported hypertension were factors significantly associated with cataract among older adults in Ghana as in Table 3. With increasing age, the risk of cataract was highest in 60–69 years (AOR = 7.8, CI 4.424-13.684 ), and slightly higher in the ≥ 70 years (AOR = 2.5, CI 1.551- 4.125) compared to older adults 50–59 years. High income (Q4 and Q5) was shown to be associated with a lower risk for self-reported cataract (AOR = 0.6, CI 0.415-0.983 ) compared to the low income group (Q1 and Q2). Older adults who did not self-report hypertension had lower risk for self-reported cataract (AOR = 0.4, CI 0.248-0.625).

**Table 3:** **Factors associated with self-reported cataract in older adults in Ghana, SAGE Wave 1**

Characteristic	Odds ratio		95% Confidence interval	P-value
		Lower	Upper	
**Sex**				
Male	---			
Female	1.364	0.77	2.42	0.287
**Location**				
Urban	---			
Rural	.926	0.59	1.44	0.733
**Age group**				
50-59 years	---			
60-69 years	7.781	4.42	13.68	0.001
70 years and above	2.530	1.55	4.13	0.001
**Educational level**				
Primary completed or less	---			
Secondary or higher	1.371	0.88	2.14	0.164
**Marital status**				
Never married	---			
Living with partner (currently married				
and cohabiting)	0.190	0.02	1.76	0.143
Living without partner (separated/divorced and widowed)	0.467	0.10	2.19	0.334
**Income status**				
Low income level (Q1 and Q2)	---			
Middle income level (Q3)	2.502	0.55	11.41	0.236
Higher income level (Q4 and Q5)	0.639	0.42	0.98	0.042
**Self-reported Stroke**				
No	---			
Yes	1.124	0.38	2.00	0.754
**Self-reported Diabetes**				
Yes	---			
No	1.169	0.52	2.61	0.703
**Self-reported hypertension**				
Yes	---			
No	0.394	0.25	0.63	0.001
**Lifetime Tobacco use**				
Ever	---			
Never	1.187	0.68	2.06	0.543
**Lifetime alcohol**				
Ever				
Never	0.879	0.55	1.40	0.587
**Level of subjective well being/life satisfaction**				
Satisfied	---			
Indifferent	.381	.046	3.14	0.370
Dissatisfied	.328	.040	2.70	0.299

## Discussion

Cataract is one of the major conditions chosen by the global initiative, Vision 2020—The Right to Sight, due to the magnitude of its contribution to the burden of blindness [22]. Several studies which focused on the burden of visual loss from cataracts demonstrate the prevalence of cataracts increases with increasing age and is slightly higher among women [23–25]. Our study confirms the findings from these studies.

An appreciation of the impact of visual impairment or blindness on one’s functional ability and quality of life is useful in providing a comprehensive picture of the burden of visual impairment beyond clinical evaluation [26]. In addition, other non-communicable and chronic diseases currently accounting for 60% of deaths globally per annum and 47% of the global burden of disease [27] are mostly associated with aging. The frequency of these conditions increases with age, and they do occur concurrently with cataract as shown in this and other studies [28, 29].

Prevalence of cataract among older persons was determined through a question on whether they have been told by a health professional that they have cataract. In the health delivery system of Ghana, these health professional are either doctors or ophthalmic nurses with capacity to diagnose basic eye conditions including cataracts [16]. The observation suggests that those diagnosed might have accessed eye care services at health facilities or during screening programs. Thus increased prevalence may imply improved access to health care and not necessarily increased disease burden. The prevalence of cataract in this group of people (5.4%) is lower than that reported in the cataract prevalence study among those 40 years and older in the Volta region, one of the ten regions in Ghana (9%) [3]. These differences may be as a result of the differences in the study design, the earlier studies were based on population screening and limited to cataract causing low vision or blindness. In this analysis most cases of cataracts (of any degree of maturity), were more likely to have been diagnosed by trained health professionals at the health facility; hence the relatively higher prevalence in the earlier studies.

Urban residents reported statistically significant higher rates (6.6%) than rural residents (4.4%). This may be related to better access to eye health care with aggregation of eye care services in urban areas in Ghana. Rural areas have limited access and residents depend mainly on erratic outreach services for eye health care. Most rural residents access health care at sub-district levels (health centres and community-based health care) where routine eye care services are unavailable. In addition overall cataract surgical uptake among older persons in Ghana from the national report on SAGE wave 1 was 48.9% [17]. Access to eye care services is critical for early risk detection and prevention of blindness or visual impairment. Well structured outreach eye care services for rural residents or inclusion of basic eye health services at sub-district levels is critical to improve eye health of older persons in Ghana.

Older adults living without a partner (separated/divorced/ widowed) reported significant higher prevalence of cataract (6.6%), compared to those with a partner (married/cohabiting) (4.3%). It may imply this group of older persons might have assessed health care previously and been diagnosed or that older persons living alone are less likely to access health care services; thus the relative higher prevalence of cataract. This may be related to other social support factors; the older adult with visual disability may have multiple disabilities as well [29]. These older adult would need more assistance (physical, social and economic) to access eye care services and living alone may limit the availability of this assistance.

Persons with higher education, higher income and health insurance reported significant higher prevalence of cataract. A potential explanation is their ability to access eye care services due to better knowledge on availability of these services, ability to afford other personal costs involved in seeking health care and improved financial access to health care through the national health insurance scheme. Older adults with low educational background on the other hand may not attach much importance to seeking early and appropriate eye care services. The fifth round of the Ghana living standards survey report in 2008 showed that 31% of all adults had never been to school, less than one-fifth (17%) attended school but did not obtain any qualifications; while a small percentage of 14% possessed secondary or higher qualification [30]. Improving the literacy level of Ghanaians is likely to encourage self-reporting to medical facilities for early diagnosis and treatment.

Generally, in this analysis, older persons in low income groups, poorly educated or living alone had some difficulty seeking vision services and care to prevent blindness or visual impairment. This constitute a vulnerable group who may require special attention to access eye care services. Social protection strategies including enrolment of older adults freely on the national health insurance scheme (NHIS) should be a national health policy worth considering. Currently only older persons ≥ 70 years are eligible for free enrolment on the NHIS [31].

Analysis also demonstrated that prevalence of self-reported cataract was relatively higher in older persons with health related factors such as obesity, diabetes, hypertension, previous history of stroke and alcohol use. Prevalence of cataract in persons with obesity, diabetes, hypertension, previous history of stroke were 5.1%, 8.4%, 10.4% and 11.4% respectively. Among older persons who had ever used alcohol or tobacco, the prevalence was 5.7% and 4.9% respectively. These findings agree with those in other studies that found similar associations [23, 24]. An interesting observation however, was that older persons who had used tobacco had relatively lower prevalence of self-reported cataract. The authors suggest a more detailed study to determine possible factors that may account for this.

A public health approach on behavioural modification (for modifiable and preventable risks- obesity, alcohol consumption and tobacco use) may be required to improve the eye health of older persons in Ghana. Diabetes is recognized as a significant risk factor due to it metabolic effect on the crystalline lens [32, 33]; disease specific health prevention efforts at national and local levels is imperative.

Analysis on satisfaction with daily living among older persons indicate prevalence of cataract is highest among the few older persons who expressed dissatisfaction with life (9.9%) and is lowest (4.7%) among those satisfied with life. Studies among older persons in communities, identified hearing and visual impairments as important factors that lead to functional decline and increased morbidity (imbalance, hip fracture, and depression) as well as mortality [29]. In addition, visual loss in older adults poses significant challenges for families who have to support them; existence of such social support for older persons in communities is often lacking [29].

In Bangladesh, Polack and others in 2008 [27] found that cataract related visual impairment negatively affected perceived health and well-being, beyond vision-specific experience. Also worsening general functioning, psychosocial and overall eyesight scores were found to be associated with increased visual loss. In their study, those with self-reported cataract significantly reported more problems with mobility, self-care, activities of daily living, pain and depression/anxiety and had significantly poorer mean self-rated health. Influence of cataract on the health and social wellbeing of individuals have also been demonstrated in studies from Kenya [14] and India [13].

### Limitation

Calling other binding diseases as cataract, and labelling any ocular/orbital/adnexal surgical procedures as cataract surgery by the older adults are very likely. The SAGE Wave 1 relied on individual submissions and did not objectively confirm cataract diagnosis or cataract surgery. The self-report of health conditions, (such as cataract and hypertension), is likely to result in underestimation of prevalence rates compared to measured rates [34]. The analysis however, provides information on prevalence of self-reported cataract among older persons across the regions of Ghana as a baseline for further investigations.

## Conclusion

Prevalence of cataract increased significantly with increasing age, was slightly higher among women, urban residents and older adults living without a partner. Significantly higher prevalence of self-reported cataract were observed in older persons with higher education, higher income and health insurance (due probably to better access to eye health care). Those who were poorly educated and with low incomes had some difficulty seeking vision services and care to prevent blindness or visual impairment.

Risk modification through effective primary prevention and health promotion efforts; behavioural modification, including public health campaigns are key efforts to limit the burden of cataract among older persons in Ghana. Well structured national outreach eye care services for rural residents and inclusion of basic eye health services at sub-district health levels of Ghana's primary health care structure are needed. Increased family support and national social protection strategies (including enrolment of older adults on national health insurance scheme) linked to the national aging policy will improve access to eye health care.

## Authors’ information

Yawson AE , Mensah G, MinicuciN and Biritwum RB are members of the WHO Multi-country SAGE Team who conducted the SAGE Survey in Ghana. Naidoo N, Chatterji S and Kowal P are members of the WHO Multi-country SAGE Team and coordinators of the multi-country study at the WHO Headquarters in Geneva. EM Ackuaku-Dogbe is a Consultant Ophthalmologist of the University of Ghana Medical School and NA Hagan Seneadza is a Specialist Public Health Physician of the Korle-Bu Teaching Hospital.
